# *Campylobacter fetus* prosthetic valve endocarditis presenting as a stroke

**DOI:** 10.1099/jmmcr.0.005147

**Published:** 2018-04-06

**Authors:** Christina Petridou, Lenka Strakova, Ruan Simpson

**Affiliations:** Microbiology Department, Queen Alexandra Hospital, Southwick Hill Rd, Cosham, Portsmouth PO6 3LY, UK

**Keywords:** *Campylobacter fetus*, prosthetic valve endocarditis

## Abstract

**Introduction:**

*Campylobacter* is a common pathogen of the gastrointestinal tract, but invasive disease is rare. *Campylobacter fetus* can play a role in osteomyelitis, meningitis and joint infection and has a prediliction for the vascular endothelium, causing mycotic aneurysms, thrombophlebitis and endocarditis. Here we present a case of prosthetic valve endocarditis caused by *C. fetus* and a review of the literature.

**Case presentation:**

An 85-year-old woman with a tissue aortic valve replacement and atrial fibrillation was admitted to hospital with tonic-clonic seizures, right-sided hemiparesis, facial droop and hemianopia. Multiple cerebral emboli were seen on magnetic resonance imaging of the brain. Blood cultures grew *C. fetus* and an echocardiogram showed thickening and restricted movement of the aortic valve, a significant difference from an echocardiogram done 2 months before when the same organism was again isolated in blood cultures. She improved after treatment with 6 weeks of amoxicillin and 2 weeks of synergistic gentamicin for prosthetic valve endocarditis.

**Conclusion:**

There have only been five previously reported cases of *C. fetus* prosthetic valve endocarditis and this is the only patient who presented as a stroke. The majority of surviving patients required replacement of the affected valve with only one other patient surviving in the absence of surgery. No guidelines exist on the optimum treatment of *C. fetus* endocarditis and this case reports adds to the growing literature on the appropriate management for this rare and potentially devastating disease.

## Introduction

*Campylobacter fetus* infection in humans is rare, but can be invasive with a fatality rate of 14 % reported in such cases and is the most common *Campylobacter* species causing bacteraemia [1]. Predisposing factors include immunosuppression, pregnancy, older age, medical device implants and cardiovascular disease with valve abnormalities [1, 2]. Most infections manifest as an acute diarrhoeal illness but the clinical presentation can be diverse, including an undifferentiated fever, meningitis, meningoencephalitis, osteomyelitis, prosthetic joint infections and lung abscesses [1]. *C. fetus* has a prediliction for vascular endothelium, causing mycotic aneurysms, thrombophlebitis and endocarditis including infections of prosthetic heart valves. The morbidity and mortality for *C. fetus* prosthetic valve endocarditis is high and no guidelines exist regarding its management, with only a handful of cases having been previously published. Here we share our experience in the management of *Campylobacter* endocarditis by presenting a case of prosthetic valve endocarditis caused by *C. fetus* and a review of the literature

## Case report

An 85-year-old woman presented to hospital on 10 July with shortness of breath, fever and raised inflammatory markers and she was treated with levofloxacin for possible pneumonia. Her past medical history was notable for a tissue aortic valve replacement 11 years prior to this event, she had atrial fibrillation and was on apixaban and had hypertension and hypothyroidism.

Blood cultures taken 2 days after admission were negative and a computed tomography pulmonary angiogram showed bibasal pleural effusions thought to be parapneumonic in nature. Deterioration in her clinical condition and new febrile episodes prompted repeat blood cultures to be taken on 28 July and these were positive for *Campylobacter fetus* identified by matrix-assisted laser desorption ionization time-of-flight analysis after 3 days of incubation. She did not have any loose stools during her admission at any point and had no known contact with animals. She was treated with 3 days of azithromycin. A transthoracic echocardiogram (TTE) was done and was reported as showing the prosthetic aortic valve was well seated with a preserved ejection fraction and mild diastolic dysfunction. She was discussed with the Cardiology team who felt that she did not warrant any further investigation as she did not display any features of endocarditis and her inflammatory markers had normalized, and she was discharged on 25 August.

She was then re-admitted on 6 September with multiple tonic-clonic seizures. In between episodes it was noticed she had a unilateral facial droop and had developed a fixed gaze. She was afebrile with a heart rate of 116 bpm and a blood pressure of 127/80 mmHg, and on examination it was noted that she had an audible ejection systolic murmur. Her C-reactive protein (CRP) level was 23 mg l^−1^ on admission and she had a white cell count of 13.0 g l^−1^ in the context of recurrent seizures.

She was initially started on ceftriaxone 2 g twice daily and acyclovir as there were concerns regarding central nervous system infection. However, as she slowly began to recover it became evident that she had a persistent right-sided facial droop, hemianopia and right-sided hemiparesis. An electroencephalogram done on admission was consistent with a vascular insult in the left hemisphere and not encephalitis and ceftriaxone and acyclovir were stopped. An initial computed tomography (CT) scan showed severe small vessel disease and a magnetic resonance imaging (MRI) scan done on 10 September showed bilateral emboli in both cerebral hemispheres. The working diagnosis at this point was that the patient had a cerebrovascular event secondary to her atrial fibrillation and she was transferred to the stroke team.

Over the next 2 weeks her inflammatory markers started to climb and she started spiking temperatures to over 38 °C on 22 September and became less rousable. Aspiration pneumonia was suspected clinically and she was started on co-amoxiclav. Blood cultures were taken and were again positive 2 days later with *C. fetus*. As previously, the patient had no gastrointestinal symptoms. At this point clarithromycin was added pending MICs.

Given the repeatedly positive blood cultures, possibly septic emboli seen on MRI scan and prosthetic heart valve, endocarditis was strongly suspected. A repeat TTE on 26 September showed a significant change in the aortic valve appearance compared to the previous imaging, with thickening and restricted movement with possible thickening of the aortic root and an increase in the peak gradient to 67 mmHg although systolic function was maintained. It was felt that this warranted treatment for infective endocarditis due to *C. fetus.* A CT angiogram showed no evidence of aortitis but a possible wedge perfusion defect was noted in the spleen. She was not considered a surgical candidate and given her age, frailty and co-morbidities a transoesophageal echo was not performed.

MICs were determined and were as follows: amoxicillin 0.50 µg ml^−1^, meropenem 0.016 µg ml^−1^, azithromycin 0.125 µg ml^−1^, gentamicin 0.38 µg ml^−1^, tetracycline 0.75 µg ml^−1^ and ceftriaxone 3 µg ml^−1^. Based on this information the antibiotics were switched to intravenous (IV) amoxicillin 2 g every 4 h to complete 6 weeks for prosthetic valve endocarditis along with 2 weeks of synergistic gentamicin at 1 mg kg^−1^ twice daily. Repeat blood cultures taken on antibiotic treatment were negative.

The patient gradually improved, her temperatures normalized and her CRP dropped to 7mg l^−1^ . She remained in hospital to complete her antibiotic treatment and multiple repeat blood cultures were negative. She remained afebrile with normal inflammatory markers while in hospital for 3 weeks after completing her treatment and was discharged to a rehabilitation facility on 28 November. There was no evidence of relapse of infection 2 months after discharge.

## Discussion

*Campylobacter* species are small, Gram-negative, curved bacteria that are motile. *C. fetus* differs from most other pathogenic species because it grows at 25  and 37 °C but not at 42 °C [3, 4], under microaerophilic conditions. Like other *Campylobacter* species, it is oxidase-positive. The primary reservoir for *C. fetus* is the gastrointestinal tract of sheep and cattle and several case reports exists of infection following ingestion of raw meat or a history of farm or animal exposure [1].

The majority of *Campylobatcer* infections are caused by *C. jejuni* and *C. coli* and normally present as a self-limiting gastrointestinal illness, although bacteraemia occurs in 0.15 % of cases [4]. In national surveillance in the US, *C. fetus* represented only 0.3 % of all *Campylobacter* species isolated from clinical samples and most of these were from blood and in the setting of bacteraemia. *C. fetus* accounts for 19–53 % of *Campylobacter* bacteraemias [4]. In the adult population the male to female ratio was 3 : 1 [5].

*C. fetus* has a predilection for the vascular endothelium, which may be explained by the presence of a surface (S)-layer protein that functions as a capsule, inhibiting C3 binding and making the organism resistant to the bactericidal activity of human serum, thereby allowing bacteraemia to occur [6]. Endocarditis due to Gram-negative bacteria remains a rare phenomenon, but infection with non-HACEK (*Haemophilus* species, *Aggregatibacter* species, *Cardiobacterium hominis*, *Eikenella corrodens* and *Kingella* species) organisms is becoming more common [4]. Numerous cases of *C. fetus* native valve endocarditis have been reported in the literature, with the majority affecting the aortic valve and occurring in male patients with previous valvular heart disease [6]. A PubMed search using the keywords ‘*Campylobacter fetus*’, ‘prosthetic’ and ‘endocarditis’ identified only five previous published cases of *C. fetus* prosthetic valve endocarditis over the past 30 years that are summarized in Table 1.

**Table 1.** Cases of *Campylobacter fetus* prosthetic valve endocarditis

Author	Sex	Age (years)	Valve	Valve age (years)	Co-morbidities	Exposure	Presentation	Antibiotics	Valve replacement surgery	Outcome
Caramelli *et al*. [8]	Female	48	Mitral	9	NK	NK	Fever, weight loss	IV pen 42 days plus IV streptomycin 13 days then IV gent for the remaining 29 days	Yes	Survived
Farrugia *et al*. [3]	Female	76	Aortic	3	NK	NK	Fever, weight loss, lethargy	IV amox 22 days plus gent 15 days then PO cipro (unclear duration)	Yes	Survived
Peetermans *et al*. [9]	Male	61	Aortic	26	Coronary artery disease, gastric ulcer	NK	Fever, malaise, weight loss	IV erythromycin and IV gent	No	Died
Haruyuma *et al*. [10]	Female	65	Aortic	5	NK	Raw meat, dental caries	Fever, sore throat, tooth ache. Readmitted with fever and new vegetation	IV amp 6 weeks plus IV gent 2 weeks then PO amox 4 weeks then IV imipenem/cilastatin plus IV gent 4 weeks then PO amox (unclear duration)	No	Survived
Reid *et al*. [4]	Male	Late 70s	Aortic	4	Hypertension, high cholesterol	Steak tartare, dental cleaning	Fever, chills, night sweats. Readmitted with septic emboli and confusion	IV ceftriaxone 3 days then IV meropenem 6 weeks then IV ertapenem 6 weeks	Yes	Survived
Our case, 2017	Female	85	Aortic	11	Hypertension	None	Tonic-clonic seizures, facial droop, unilateral weakness, hemianopia	IV amox 6 weeks plus IV gent 2 weeks	No	Survived

NK, not known; Gent, gentamicin; Pen, penicillin; Amox, amoxicillin; Cipro, ciprofloxacin; Amp, ampicillin; IV, intravenous; PO, oral.

Of the six cases of prosthetic valve endocarditis, all but one occurred in patients over 60 years and four of the cases were females. The aortic valve was affected most frequently with only one case affecting the mitral valve, with the valve age ranging from 3 to 26 years. Two of the patients had exposure risks in the form of eating raw meat, which has been reported in several other case reports of native valve *C. fetus* endocarditis. There was no known farm or animal exposure identified in our case, although ingestion of undercooked meat could not be excluded. Most patients presented with typical features of endocarditis such as fever, weight loss and lethargy apart from our patient who presented with seizures and a stroke. None of the patients reported recent gastrointestinal symptoms.

No guidelines exist for the management of *Campylobacter* endocarditis and trial data to recommend the optimum treatment for patients with non-HACEK Gram-negative endocarditis is limited, with early consideration of cardiac surgery and a prolonged course of combination antibiotic therapy involving a beta-lactam plus an aminoglycoside supported by the Infectious Diseases Society of America and British Infection Association [7, 8]. Half of the six published cases of *C. fetus* prosthetic valve endocarditis required valve replacement, and one patient who did not undergo surgery died. Most of the patients in this series received a beta-lactam plus aminoglycoside combination during their treatment apart from the patient who died, and they received erythromycin and gentamicin apart from one other patient who survived who did not have a synergistic aminoglycoside added. The minimum duration of IV therapy was 22 days with the majority treated with 6 weeks IV antibiotics with some following on with a prolonged oral course.

Although *C. fetus* infection is a rare disease, the isolation of *C. fetus* in blood cultures should prompt clinicians to suspect involvement of the heart or blood vessels. There is a high mortality rate in the absence of surgery and complications may be late, including septic emboli or perivalvular extension despite adequate treatment. In this particular case, the short duration of antibiotic treatment on first presentation may have been a factor in the patient presenting with complications of endocarditis. Early involvement of infection specialists is imperative in managing these patients to obtain an antibiotic regimen, with treatment choice relying on interpreting laboratory data and the combination treatments chosen being associated with significant toxicities and needing careful monitoring. This case of prosthetic valve endocarditis adds to the growing literature on this rare disease and its management.
